# Association between COX2 -765G/C polymorphism and periodontitis in Chinese population: a meta-analysis

**DOI:** 10.1186/s12903-018-0483-9

**Published:** 2018-03-07

**Authors:** Zhan-shan Zhang

**Affiliations:** Department of Stomatology, the First Hospital of Hohhot, Hohhot, Inner Mongolia 010030 China

**Keywords:** Cyclooxygenase-2, Polymorphism, Periodontitis, Meta-analysis

## Abstract

**Background:**

Studies had attempted to clarify the relation between COX2 -765G/C gene polymorphisms and periodontitis risk, but there has been no definite consensus to date. A meta-analysis was performed to further explore the relationship of COX2 -765G/C polymorphism on periodontitis risk among Chinese population.

**Methods:**

The databases of PubMed, Springer Link, Ovid, Chinese Wanfang Databases, Chinese National Knowledge Infrastructure (CNKI) and Chinese Biology Medicine were searched up to January 2017. The overall result and subgroup analysis results were combined using fixed-effect or random-effect based on the heterogeneity.

**Results:**

Finally, 7 case–control publications including 1399 periodontitis cases and 1663 controls were identified according to the inclusion criteria. In the total analyses, COX2 -765G/C polymorphism had nonsignificant association on periodontitis risk in all models. The subgroup analyses suggested a significantly increased risk of periodontitis in studies with population-based controls and a significantly decreased risk in studies with hospital-based controls.

**Conclusions:**

This meta-analysis indicated that COX2 -765G/C polymorphism had significantly affect on periodontitis risk among Chinese individuals, which should be confirmed by other ethnic groups.

## Background

Periodontitis, divided into chronic periodontitis (CP) and aggressive periodontitis (AP), is a multifactorial disease, involving both genetic and environmental risk factors. Previous report had confirmed that periodontal disease is at least partially determined by genetic [1]. The factors by genetic, for example, interleukin gene polymorphisms [2–5], transforming growth factor gene polymorphisms [6], and vitamin D receptor gene polymorphisms [7], are known as an important role on the development of periodontitis [8]. Cyclooxygenase is a key enzyme that converts arachidonic acid to prostaglandin H2. Cyclooxygenase-2 (COX-2) increases prostaglandin in tumor tissue. COX-2 plays an important role in cell proliferation, angiogenesis, and Alzheimer’s disease [9]. The COX-2 gene includes many different polymorphic sites [10–13]. Among those, COX2 -765G/C has been the most extensively investigated with periodontitis recently, with no exact association up to now. Differences in findings may be due to race and clinical heterogeneity in patients who have been studied, as well as the limited number of cases and participants in every individual study. It is the best way to get over the small sample size problems and inadequate statistical power. In evaluating the association of COX2 -765G/C polymorphism with the risk of periodontitis in a solely Chinese population, we conducted the present updated meta-analysis to reduce the influence of the diverse genetic backgrounds. The subgroup analyses were conducted to further explore the possible relation of gene environment interaction on periodontitis risk.

## Methods

### Search strategy and selection criteria

The databases of PubMed, Springer Link, Ovid, Chinese Wanfang Data databases, Chinese National Knowledge Infrastructure (CNKI) and Chinese Biology Medicine were searched for studies examining the relation between COX2 -765G/C polymorphism and periodontitis risk up to January 2017. The search keywords were namely: (periodontitis or periodontal disease) and (cyclooxygenase-2 or COX-2) and (China or Chinese or Taiwan). No restriction was imposed on search language. In addition, we also reviewed the references cited in the searched articles to look for other related studies.

The following criteria were used: (1) they were case-control or cohort studies describing the association of the COX2 -765G/C polymorphism and periodontitis, (2) they provided the genetypes in cases and controls, (3) the participants were of the Chinese population. Exclusion criteria: (1) repeated literature, (2) incomplete data, (3) case-only articles, (4) review articles and abstracts, (5) participants with systematic diseases.

### Data extraction

The Preferred Reporting Items for Systematic Reviews and Meta-Analyses (PRISMA) statement was used in our report. Titles and abstracts of all identified studies were screened firstly. We then reviewed full articles when it is ambiguous only read to title and abstract. Data extracted from identified studies included first author’s name, publication years, type of periodontitis, the source of controls, geographical area, cases and participants, and the people with COX2 -765G/C genotypes. Hardy-Weinberg equilibrium (HWE) in controls was calculated using corresponding genotype distribution.

### Statistical analysis

The following models were used in our report: (1) allelic contrast, (2) contrast of homozygotes, (3) recessive, and (4) dominant models. The χ^2^-test was performed to determine the Hardy-Weinberg equilibrium (HWE) of genotypes. We also calculated the heterogeneity of rare allele frequencies by χ^2^-test when it is available in control groups of every individual study included. The association of COX2 -765G/C polymorphism and periodontitis risk was assessed using odds ratio (ORs) and their 95% confidence intervals (CIs). The Mantel-Haenszel’s fixed-effect model was used when the between-study heterogeneity was below 50%. Otherwise, the DerSimonian and Laird’s random-effect model was used to combine the overall ORs and 95%CIs. Significance of the pooled ORs was calculated using the Z-test. Sensitivity analysis was performed to note whether a single study could influence the overall result while removing a single study at a time. Furthermore, subgroup analyses by geographical area, source of controls, and type of periodontitis were also performed. All statistical tests were calculated by the Stata 12.0 (StataCorp LP, College Station, TX). A *p*-value less than 0.05 was considered to be significant.

## Results

### Description of included studies

**Fig. 1:**
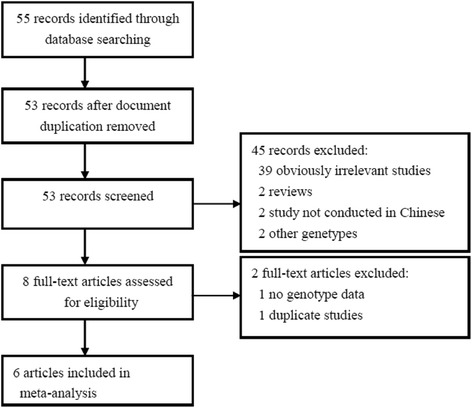
Flow diagram of the literature search

Figure 1 illustrates the literature search process in the form of a flow chart. Fifty-five related records were retrieved through database searching. When we first reviewed the titles and abstracts, 47 articles were excluded according to the exclusion criteria described. Then we reviewed all of the remaining full-articles, two were excluded due to duplication and lack of raw data. Finally, 6 articles (seven case-control studies) [14–19] were included at the end. The publication years of involved studies were from 2008 to 2012. In general, 1399 periodontitis cases and 1663 health controls were included in this report. The source of controls in four studies was population-based. The characteristics of included studies are shown in Table 1.

**Table 1:** Characteristics of studies included in the meta-analysis

References	Type of periodontitis	Source of controls	Geographic area(s)	Case number	Control number	Cases			Controls			HWE	
						GG	GC	CC	GG	GC	CC	χ^2^	*P*
Ho, 2008 (15)	CP	HB	Taiwan	343	153	242	93	8	89	60	4	2.77	0.096
Ho, 2008 (15)	AP	HB	Taiwan	85	153	81	3	1	89	60	4	2.77	0.096
Sun, 2009 (16)	CP	PB	Guangdong	150	143	119	29	2	119	21	3	2.85	0.091
Xie, 2009 (17)	CP	HB	Guangdong	146	148	137	9	0	128	20	0	0.78	0.378
Loo, 2011 (18)	CP	PB	Sichuan	280	250	134	68	78	152	90	8	1.50	0.221
Li, 2012 (19)	CP	PB	Sichuan	122	532	55	33	34	316	204	12	10.24	0.001
Duan, 2012 (20)	CP	PB	Ningxia	273	284	205	57	11	223	54	7	2.72	0.099

*PB* population-based, *HB* hospital-based, *CP* chronic periodontitis, *AP* aggressive periodontitis

### Meta-analysis

**Fig. 2:**
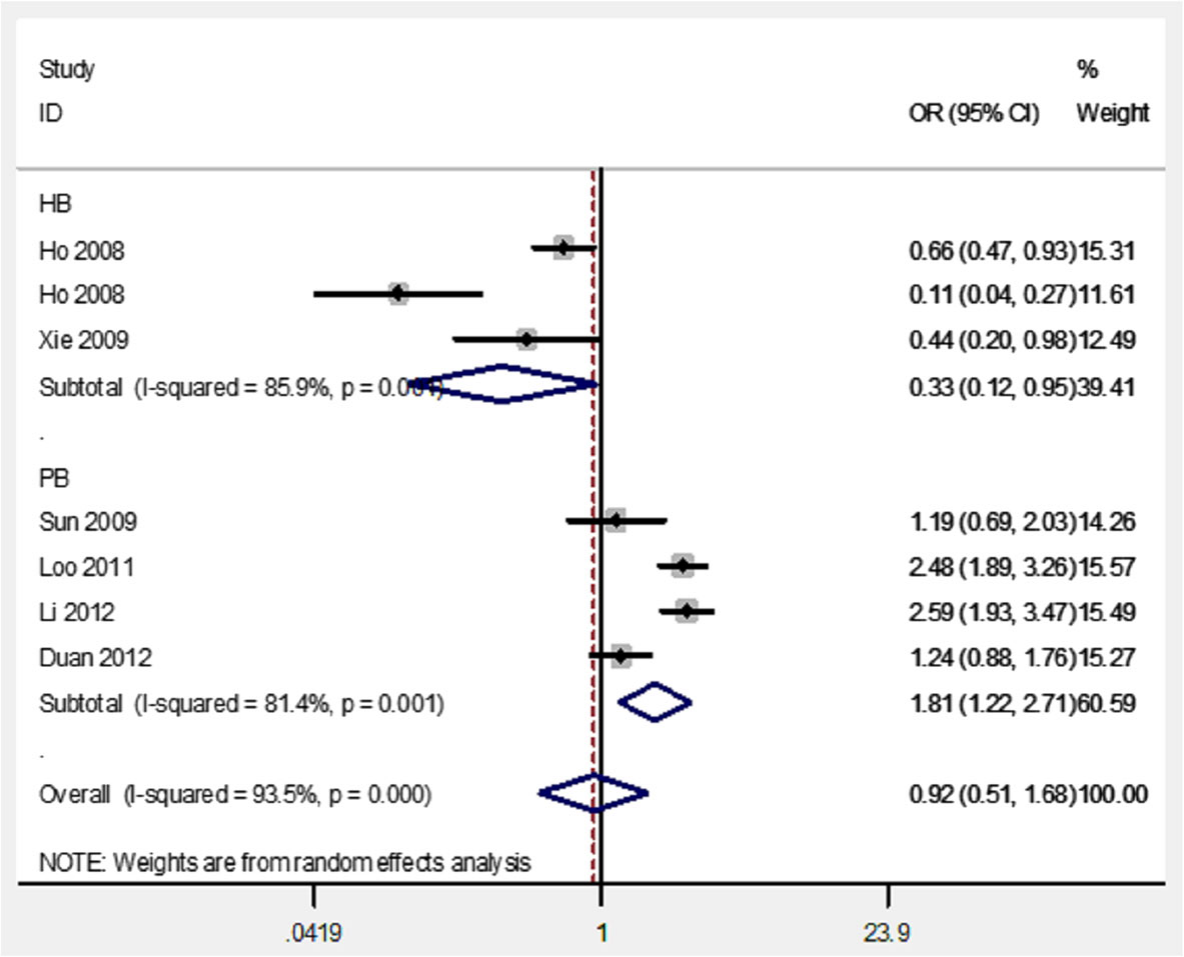
The forest plots of all selected studies on the association between COX2 -765G/C polymorphism and periodontitis risk in Chinese (for allele model)

**Fig. 3:**
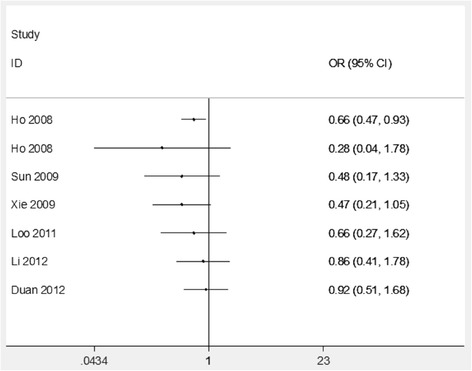
Cumulative analysis of the relationship between COX2 -765G/C polymorphism and periodontitis risk in Chinese (for allele model)

Table 2 lists the primary results in our analyses. First, a heterogeneity analysis was conducted, and there is no significant relation found between COX2 -765G/C polymorphism and the risk of periodontitis in the total analyses (Fig. 2). The cumulative analysis further indicated a lack of relation between COX2 -765G/C polymorphism and periodontitis risk in the allele model (Fig. 3). In the subgroup analyses, a significantly increased risk of the association between the COX2 -765G/C variants and periodontitis was found in studies with population-based controls (C vs. G, ORs = 1.81, CI = 1.22–2.71; CC vs. GG, ORs = 4.47, CI = 1.25–15.95; CC vs. GG + GC, ORs = 4.47, CI = 1.21–16.44; CC + GC vs. GG, ORs = 1.52, CI = 1.24–1.86), while a significantly decreased risk was found in studies that with hospital-based controls (C vs. G, ORs = 0.33, CI = 0.12–0.95; CC + GC vs. GG, ORs = 0.28, CI = 0.09–0.88) (Table 2 and Fig. 2).

**Table 2:** Association of the COX2 -765G/C gene polymorphism on periodontitis susceptibility

Analysis model		n	ORr(95%CI)	ORf(95%CI)	P_h_
C vs. G	Total analysis	7	0.92 (0.51–1.68)	1.34 (1.17–1.54)	0.000
	Hospital-based	3	**0.33 (0.12–0.95)**	**0.45 (0.34–0.60)**	0.001
	Population-based	4	**1.81 (1.22–2.71)**	**1.99 (1.69–2.35)**	0.001
	South China	6	0.86 (0.41–1.78)	1.36 (1.17–1.58)	0.000
	CP	6	1.24 (0.73–2.11)	1.54 (1.33–1.78)	0.000
CC vs. GG	Total analysis	6	2.25 (0.64–7.95)	4.82 (3.30–7.06)	0.000
	Hospital-based	2	0.58 (0.20–1.70)	0.55 (0.20–1.54)	0.441
	Population-based	4	**4.47 (1.25–15.95)**	**7.01 (4.55–10.78)**	0.000
	South China	5	2.33 (0.53–10.31)	5.77 (3.78–8.81)	0.000
	CP	5	3.08 (0.86–1.03)	5.53 (3.70–8.26)	0.000
CC vs. GG + GC	Total analysis	6	2.50 (0.73–8.52)	5.31 (3.64–7.74)	0.000
	Hospital-based	2	0.76 (0.26–2.19)	0.74 (0.26–2.07)	0.587
	Population-based	4	**4.47 (1.21–16.44)**	**7.29 (4.77–11.15)**	0.000
	South China	5	2.69 (0.66–11.05)	6.47 (4.25–9.88)	0.000
	CP	5	3.21 (0.90–11.41)	5.87 (3.95–8.73)	0.000
CC + GC vs. GG	Total analysis	7	0.79 (0.45–1.38)	1.00 (0.85–1.18)	0.000
	Hospital-based	3	**0.28 (0.09–0.88)**	**0.38 (0.27–0.52)**	0.001
	Population-based	4	**1.52 (1.24–1.86)**	**1.52 (1.24–1.86)**	0.469
	South China	6	0.71 (0.36–1.42)	0.96 (0.80–1..15)	0.000
	CP	6	1.08 (0.70–1.65)	1.18 (0.99–1.40)	0.000

*ORr* Odd ratio for random-effect model, *ORf* Odd ratio for fixed-effect model, *P*_*h*_ *P* value for heterogeneity test; North China included Ningxia; South China included Taiwan, Sichuan, Guangdong

**Fig. 4:**
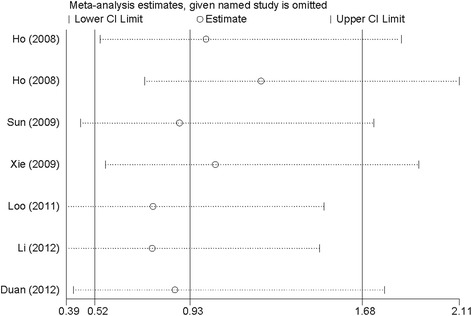
A leave-one-out sensitivity analysis to evaluate the stability of the meta-analysis

We also performed a leave-one-out analysis to explore whether the sensitivity of our meta-analysis was existence. The results were essentially unchanged while removing a single individual study at a time, suggesting that the result in our study was comparatively credible and stable (Fig. 4).

## Discussion

Periodontitis is a multifactorial disease with the risk factors including oral microorganisms [20] and tobacco use [21]. The variation among individual in susceptibility to periodontitis indicates the key role of genetic factors in their pathogenesis [8]. The relationship between COX2 -765G/C polymorphism and periodontitis risk attracted the attention of many researchers. However, results of individual studies were inconclusive. Regional and racial differences are one likely reason for the conflict results. We therefore performed this study to explore the relation between COX2 -765G/C polymorphism and periodontitis risk in a single ethnic group.

Seven case-control studies were included in this study comprising 1399 periodontitis cases and 1663 controls. Pooled result indicated that a significantly increased risk of the association between the COX2 -765G/C variants and periodontitis in studies with population-based controls and a significantly decreased risk in studies with hospital-based controls was found. Till now, there are only two meta-analyses published on the COX2 -765G/C and periodontitis [22, 23], one [22] paper showed that -765G/C variants could reduce the CP risk in the co-dominant models (GC vs. GG: ORs =0.77, 95% CI = 0.61–0.94) for Asian, while another [23] paper showed a reduced risk for CP among Chinese population with limited population. Therefore, these two studies were conducted among Chinese populations using smaller number of publications, and did not perform a subgroup analysis by source of controls. Especially for Prakash et al.’s meta-analysis [23], 2 studies performed in Chinese participants were only included, and all the controls were hospital-based. The selection bias would be occurred in hospital-based controls because such populations could not represent the general populations. Moreover, matching on social class may also be another reason for the opposite results between studies with population-based controls and hospital-based controls. These inherent matching problems need to be considered in selecting control populations for case-control studies. Furthermore, results from this study showed strong evidence between COX2 -765G/C variants and periodontitis risk in a Chinese population.

Several strengths were showed in our study. First, we strictly followed the PRISMA methodology to develop inclusion criteria and exclusion criteria to reduce the possible selection bias. Second, little publication bias and other bias would be appearance while we only included Chinese populations. Third, we investigated the influence of a geographic area and the source of control on the risk of periodontitis and COX2 -765G/C. Fourth, sensitivity analysis indicated no single study had effect on the whole result, suggesting our results are reliability and stability. Several limitations should also be considered. First, this ethnic-specific meta-analysis only included data from a single ethnic group; therefore, the result obtained in our study is only applicable to Chinese populations. Second, since this meta-analysis was based primarily on unadjusted ORs and CIs, it is not possible to control confounding factors. Third, we have devised a comprehensive search strategy, but including the number of research is still relatively small. Finally, the publication bias was not evaluated due to the limitations of funnel plot.

## Conclusions

This meta-analysis indicates a significant risk between COX2 -765G/C polymorphism and periodontitis in the Chinese population. Ethnicity and control sources seem to have a confounding effect on these studies. Based on the limited number of included studies, further studies with large cases and participants are required to confirm this result.

## Abbreviations

AP: aggressive periodontitis
CI: Confidence interval
COX-2: Cyclooxygenase-2
CP: chronic periodontitis
HWE: Hardy-Weinberg equilibrium
OR: Odds ratio
PRISMA: Preferred Reporting Items for Systematic Reviews and Meta-Analyses
